# Melatonin-mediated temperature stress tolerance in plants

**DOI:** 10.1080/21645698.2022.2106111

**Published:** 2022-08-19

**Authors:** Ali Raza, Sidra Charagh, Pedro García-Caparrós, Md Atikur Rahman, Vincent H. Ogwugwa, Faisal Saeed, Wanmei Jin

**Affiliations:** a College of Agriculture, Oil Crops Research Institute, Fujian Agriculture and Forestry University (FAFU), Fuzhou, Fujian, China; b State Key Laboratory of Rice Biology, China National Rice Research Institute, Chinese Academy of Agricultural Sciences (CAAS), Hangzhou, Zhejiang, China; c Agronomy Department of Superior School Engineering, University of Almería, Almería, Spain; d Grassland and Forage Division, National Institute of Animal Science, Rural Development Administration, Cheonan, Korea; e Department of Microbiology, University of Lagos, Nigeria; f Department of Agricultural Genetic Engineering, Faculty of Agricultural Sciences and Technologies, Nigde Omer Halisdemir University, Turkey; g Key Laboratory of Biology and Genetic Improvement of Horticultural Crops (North China), Institute of Forestry and Pomology, Beijing Academy of Agriculture and Forestry Sciences, Beijing, Peking, China

**Keywords:** Biostimulants, climate change, cold stress, crosstalk, food security, freezing temperature, genetic engineering

## Abstract

Global climate changes cause extreme temperatures and a significant reduction in crop production, leading to food insecurity worldwide. Temperature extremes (including both heat and cold stresses) is one of the most limiting factors in plant growth and development and severely affect plant physiology, biochemical, and molecular processes. Biostimulants like melatonin (MET) have a multifunctional role that acts as a “defense molecule” to safeguard plants against the noxious effects of temperature stress. MET treatment improves plant growth and temperature tolerance by improving several defense mechanisms. Current research also suggests that MET interacts with other molecules, like phytohormones and gaseous molecules, which greatly supports plant adaptation to temperature stress. Genetic engineering via overexpression or CRISPR/Cas system of MET biosynthetic genes uplifts the MET levels in transgenic plants and enhances temperature stress tolerance. This review highlights the critical role of MET in plant production and tolerance against temperature stress. We have documented how MET interacts with other molecules to alleviate temperature stress. MET-mediated molecular breeding would be great potential in helping the adverse effects of temperature stress by creating transgenic plants.

## Graphical Abstract

**Figure:**
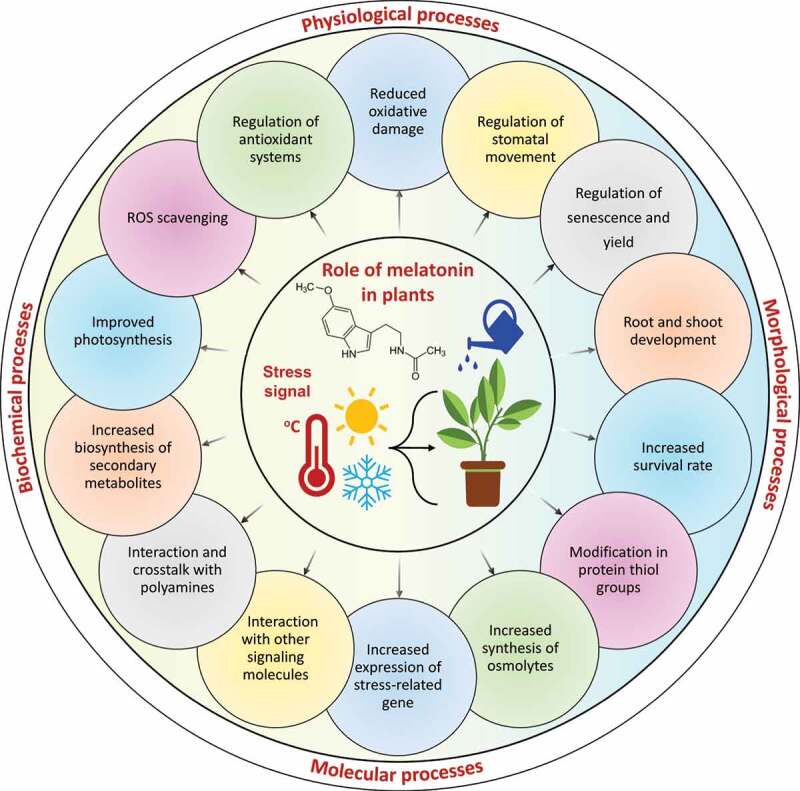

## 1. Introduction

**Figure 1.**
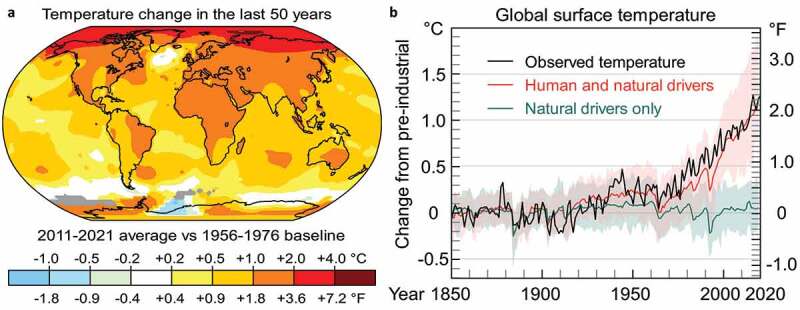
Impact of climate change on modification of environmental temperature. (a) Average surface air temperatures from 2011 to 2020 compared to the 1951–1980 average. Source: NASA (https://data.giss.nasa.gov/gistemp/, Retrieved 11 July 2022). (b) Change in average surface air temperature since the industrial revolution, plus drivers for that change. Human activity has caused enhanced temperatures, with natural forces adding some unpredictability. Source: IPCC (https://www.ipcc.ch/report/ar6/wg1/downloads/report/IPCC_AR6_WGI_Full_Report.pdf; Retrieved 11 July 2022).^2^

The influences of climate change are extremely threatening the crop production, crop phenology, plant vulnerability, and livelihood of the 2.8 billion rural peoples.^1^ While global warming and climate change increase the Earth’s air temperature (Fig. 1), negatively influencing crop growth and productivity.^2–6^ Current climate change comprises both global warming and its impacts on Earth’s climate patterns. There have been prior phases of climate change, but the present alterations are clearly quicker and not due to natural causes. Due to climate change, deserts are increasing, while heat waves and wildfires are becoming more widespread (Fig. 1).^7^ Climate warming and the subsequent rise in extreme temperatures notably augment drought incidence, interval, and strength. This expansion of drought attributes causes variations in rainfall regimes, atmospheric water vapor fluxes, and soil humidity, as well as excess and river emissions.^2,8,9^ In recent times, climate predictions warned that increases in temperature, irregular rainfall and global CO_2_ emission could have a considerable negative influences on the productivity of economically important field crops.^1,7,10^ Consequently, a better understanding on the link between crop phenology and temperature is important for exploring superior insight of biodiversity growth, natural activities, and inherent plant systems and their modifications in response to environmental factors.

Plants grow in a situation that implements various environmental factors (abiotic and biotic stresses), and discrepancy in any of these factors can hinder several morphological, physiological, and molecular variations at many steps; ultimately, plant growth and production get hampered by these stresses.^11–13^ Among different stresses, temperature extremes (i.e., cold stress (CS) such as chilling 0–15°C, and freezing <0°C, and heat stress (HS) >25°C) are causing damage to crop production and levying substantial risks to food security globally. Plants are infrequently affected by certain factors, and temperature stress alone or combined with other stresses may trigger oxidative impairment in plants. Consequently, HS and CS impede plant development as a consequence of cellular injury or even cause cell death by reducing membrane fluidity and antioxidant enzyme activities, biosynthesis of diverse proteins and secondary metabolites, and amendments in hormonal signaling after continuing exposure.^3–6,14^

Plants are required to breed and flourish to prolong their survival under temperature stress. Thus, there are numerous advances for retaining a balance between plant growth, development, and stress tolerance.^5,6,11^ To cope with temperature stress, plants modify their morphology, physiology, biochemistry, and gene expression, which alters their growing activities to grow and tolerate HS and CS conditions.^5,6,11,15^ Temperature extremes (HS and CS) can cause overgeneration of reactive oxygen species (ROS) *viz* singlet oxygen (^1^O_2_), hydroxyl (OH) radicals, superoxide (O_2_^−^), and hydrogen peroxide (H_2_O_2_).^16,17^ Especially, ROS are exceptionally sensitive to cellular objects and can cause critical oxidative impairment to metabolic events and thus restricting plant growth and development under temperature stress.^17–19^

Due to the sessile nature of plants, they are unable to escape temperature stress merely by moving to an appropriate stress-free environment. Therefore, plants have evolved a series of mechanisms to cope with undesirable stress conditions by modifying their developmental, physiological, biochemical, and molecular processes to changing environments.^5,20^ To improve plant growth and productivity under stress conditions, chemical approaches, i.e., exogenous treatment with several substances of both natural and synthetic origin, like phytohormones, osmolytes, gaseous molecules, etc., are getting much attention.^21–23^ These substances help in mitigating the adverse effect of stress conditions.

Among various substances, melatonin (MET) is considered as a master regulator. MET is an indole compound derived from serotonin (5-hydroxytryptamine); it was first discovered in animal tissues playing an important role in multiple physiological responses.^24^ MET mediates diverse functions, including organogenesis, flowering, photosynthesis, reproduction, circadian rhythm, fruit ripening and plant acclimatization to changing environments.^25^ Recent evidence on MET studies highlights the beneficial role of MET in plant stress tolerance and is considered as a regulatory hub of phytohormones.^26–28^ So, MET appears to be multi-regulatory molecules, as small molecular weight indoleamine (also known as a natural antioxidant) that takes part in various abiotic stresses, including HS and CS.^24,29–31^ Plants’ introduction to HS/CS triggers a rise in endogenous MET levels, resulting in up-regulation of MET biosynthesis genes and increased MET content. Exogenous MET improves plant tolerance against HS and CS in two aspects, i.e., either by directly scavenging ROS molecules or indirectly by improving antioxidant enzyme activities, photosynthetic efficacy, metabolite contents, and up-regulating the expression levels of MET-related and stress-inducible genes in plants.^24,29,31–34^ Thus, firstly we discussed MET biosynthesis and metabolism in response to stress conditions. The beneficial role of exogenous MET in understanding how plants respond, adapt and tolerate temperature stress has been widely explained. MET crosstalk with other molecules has also been documented under HS and CS conditions. Finally, the genetic engineering of MET-related genes has also been highlighted, indicating that the modifications in MET biosynthesis and endogenous levels can significantly improve the temperature stress tolerance by rising transgenic plants.

## 2. Metabolism and Biosynthesis of Melatonin in Plants

MET (N-acetyl-5-methoxytryptamine) was firstly isolated in 1958 in the bovine pineal gland.^35^ MET is an indolic compound derived from serotonin whose biosynthetic pathway has been widely investigated in animals and plants.^32,33^ MET has a potent natural antioxidant capacity which justifies its exogenous supply in plants subjected to stressed conditions biostimulating the plant growth under adverse conditions.^24,32,33^

**Figure 2.**
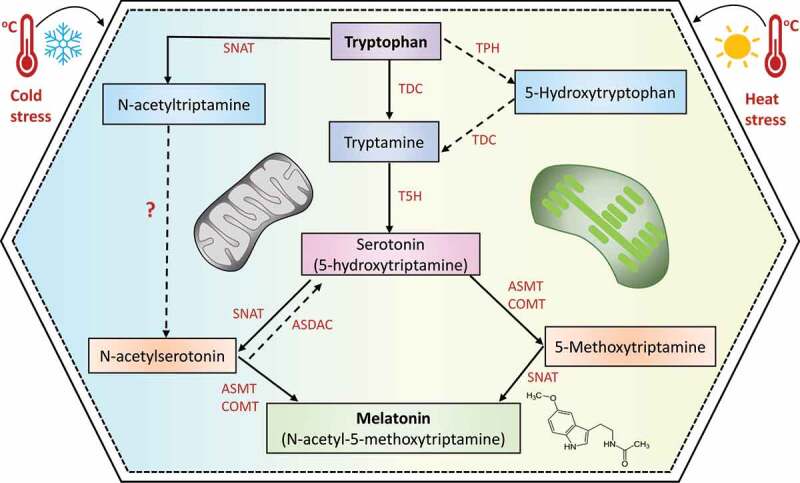
Biosynthetic pathway of melatonin in plants. The enzymes participating in this metabolism are tryptophan decarboxylase (TDC), tryptophan hidroxylase (TPH), tryptamine 5-hydroxylase (T5H), serotonin N-acetyltransferase (SNAT), N-acetylserotonin methyltransferase (ASMT), caffeic acid O-methyltransferase (COMT), and N-acetylserotonin deacetylase (ASDAC). Black arrows (TDC-T5H-SNAT-ASMT/COMT or TDC-T5H-ASMT/COMT-SNAT) are the common pathways; the dotted arrows (TPH-TDC) represent an uncommon pathway; the dotted arrow (ASDAC) represents a reverse pathway, and the dotted arrows (SNAT-?) represent the uncompleted pathway.

The metabolic biosynthesis in plants has a higher complexity than in animals. It is also necessary to point out that plants are able to synthesize more amounts of this biomolecule than animals.^36^ In plants, mitochondria and chloroplasts are the main organelles in which MET is synthesized (Fig. 2).^37^ The main site of MET biosynthesis is the chloroplasts, but if the chloroplast pathway is disrupted, the mitochondrial pathway will be energized for MET generation to preserve homeostasis.^36,37^ The levels of MET in plants can vary from picograms to micrograms per gram of tissue, depending on the tissue and the crop analyzed. Therefore, higher concentrations supplied, such as 100 mM are not desirable since it may result in toxic effects.^29,36,37^

Mainly, size enzymes are involved in the conversion of tryptophan to MET, including tryptophan decarboxylase (TDC), tryptophan hidroxylase (TPH), tryptamine 5-hydroxylase (T5H), serotonin N-acetyltransferase (SNAT), N-acetylserotonin methyltransferase (ASMT), and caffeic acid O-methyltransferase (COMT) (Fig. 2).^29,34^ The starting material for MET biosynthesis in plants is tryptophan, generated via the shikimic acid pathway.^38^ The biosynthesis of MET in plants can be achieved using two different pathways, which are activated under different conditions. For instance, under normal conditions, tryptophan is converted to MET through catalysis by TDC, T5H, SNAT, and ASMT. Nevertheless, under senescence, the catalytic process for the conversion of tryptophan to MET is carried out through TDC, T5H, ASMT, and SNAT.^39^

There are four-step reactions involved in MET biosynthesis.^39,40^ The first-step reaction is the decarboxylation of tryptophan into tryptamine through TDC. One rate-limiting step in MET biosynthesis is the participation of TDC.^41^ The second-step reaction is the hydroxylation of tryptamine via the cytochrome P450 enzyme T5H to generate serotonin.^39^ It is necessary to point out that besides the conversion of tryptophan to serotonin via TDC and T5H, there is another pathway reported in St. John’s wort (*Hypericum perforatum*) in which tryptophan is converted to 5-hydroxytryptophan through TPH and then by TDC to generate serotonin.^42^ Once generated serotonin, there are two pathways to catalyze the conversion to MET. One of these pathways is the conversion of serotonin to N-acetylserotonin via serotonin-N-acetyltransferase, which is then converted to MEL through the catalytic process via N-acetylserotonin methyltransferase (ASMT) or caffeic acid O-methyltransferase (COMT) (pathway known as NM). The second pathway is based on the conversion of serotonin to 5-methoxytryptamine catalyzed by ASMT/COMT, and finally, the synthesis of MET via SNAT (pathway known as MN).^43,44^

To describe biosynthesis and mode of action of MET, in rice, it was reported that the presence of a reverse MET pathway where N-acetylserotonin is converted to serotonin through N-acetyl- serotonin deacetylase (ASDAC).^45^ The mode of action of MET in plants is dependent on the subcellular localization of the enzymes involved. For instance, SNAT is present in mitochondria and chloroplasts. The cytoplasm possesses TPH, ASMT/COMT, and TDC, while T5H is present only in the endoplasmic reticulum.^46,47^ Considering this fact, some authors have reported that N-acetylserotonin is generated in chloroplast and then moved to the cytoplasm for O-methylation to synthesize MET. Conversely, other authors have noted that the methoxylation of serotonin to produce methoxytriptamine occurs in the cytoplasm. Then, this molecule is transported to the chloroplast for acetylation by SNAT to generate MET.^36,45^

## 3. Potential of Exogenous Melatonin in Managing Temperature Stress in Plants

**Figure 3.**
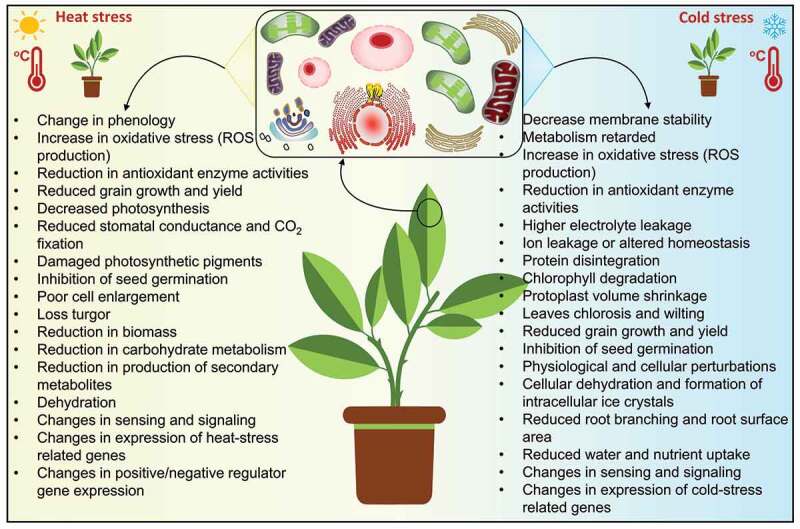
An overview of the impact of temperature stress on plant’s morphological, physiological, biochemical, molecular and cellular processes.

**Figure 4.**
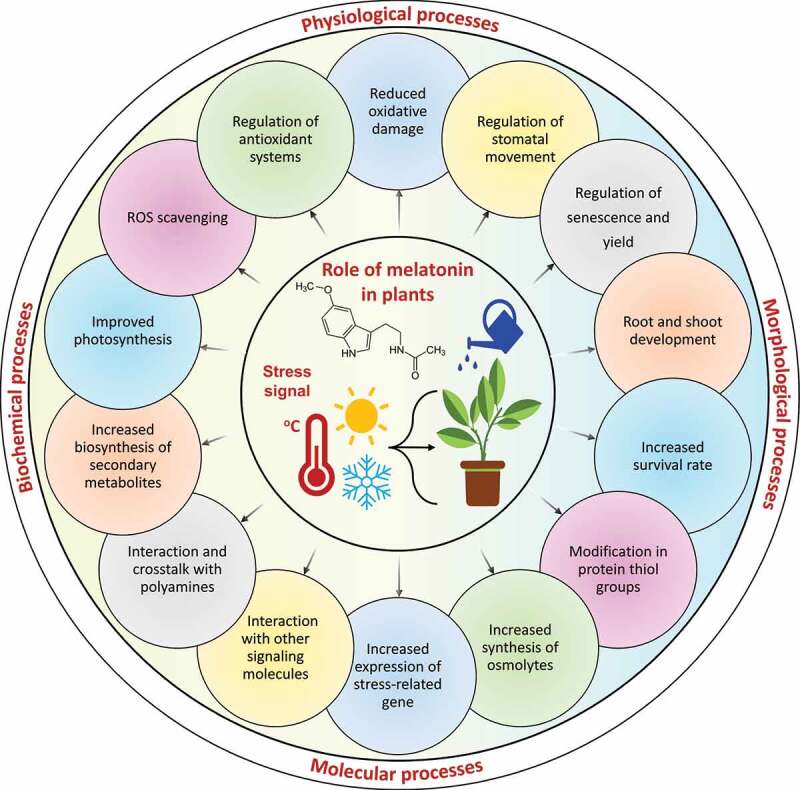
An overview of the beneficial role of melatonin in plants under temperature stress. Melatonin mainly improves several physiological, biochemical, morphological, cellular, and molecular processes and plants’ survival against stressful environments. Within the circle, the small chemical structure represents melatonin.

The plant needs an ideal temperature for normal growth and development. Changing temperature can inhibit plant growth and severely affect its developmental mechanisms. Especially, CS and HS devastatingly conquer plant growth and development by unsettling several morphological, biochemical, physiological, molecular and cellular processes (Fig. 3). Therefore, plants have evolved several MET-induced physiological, biochemical, and molecular processes to respond, adapt and attain tolerance against temperature stress (CS and HS) (Fig. 4). In the below segments, we have largely studied the defensive role of exogenous MET application in improving the adverse effect of temperature stress in various plant species.

### 3.1. Cold Stress

Cold stress (CS; including chilling and freezing temperatures) exerts multiple effects and leads to changes in biochemistry, physiology, and molecular biology in plants (Fig. 3).^4,5,48^ Cold stress also affects the photosynthetic machinery inducing photo-inhibition at both PSI and PSII.^4,5,14,48,49^ Melatonin (*N*-acetyl-5-methoxytryptamine), first reported in plants in 1995, protects plants against various environmental stresses, including CS (Table 1).^57^ In major crops, fruits and vegetables, cold stress responses are linked with both unique as well as common elements between chilling and freezing temperatures. As the optimum chilling and freezing temperature ranges vary from crop to crop, fruit to fruit and vegetables to vegetables, the following sections have given an overview of the protective roles of exogenous MET in plants under temperature stresses.

**Table 1.** Summary of some recent examples of melatonin-mediated temperature stress tolerance in different plants.

Plant specie	Stress condition	Dose	Protective role	References
**Cold stress**				
Barley (*Hordeum vulgare* L.)	2 ± 0.5°C	1 mM	Exogenous MET application increased the drought priming-induced cold tolerance (DPICT) by modulating subcellular antioxidant systems and ABA levels	^50^
Maize (*Zea mays* L.)	5°C, 14 d	50 and 500 μM	MET pretreatment-induced modifications improve the respiratory/energetic metabolism of the conditioned seeds, and these changes could be crucial for efficient stress amelioration	^51^
Cucumber (*Cucumis sativus* L.)	10°C, 7 d	50 and 500 μM	MET pretreatment improved the antioxidant defense, especially SOD and GSSG-R, stimulating glutathione’s *de novo* synthesis and augmenting other antioxidants’ activities (GSH/GSSG ratio)	^52^
*Elymus nutans*	4°C	0, 1, 10, 50, 100, 300 μM	MET application improved ABA production and up-regulated the CS-responsive genes expression in an ABA-independent manner	^53^
Rice (*Oryza sativa* L.)	12°C	20 or 100 μM	MET pretreatment relieved the stress-induced inhibitions to photosynthesis, and PSII activities and also increased the antioxidant enzyme activities and non-enzymatic antioxidant levels	^54^
Watermelon (*Citrullus lanatus* L.)	4°C	50, 150, 300, 500, or 800 μM	MET induced cold tolerance via long-distance signaling, and such induction was associated with an enhanced antioxidant capacity and optimized defense gene expression	^55^
Cucumber (*Cucumis sativus* L.)	15/8°C day/night	200 μM	MET alleviated chilling stress in cucumber seedlings by up-regulation of *CsZat12* and modulation of polyamine and abscisic acid metabolism	^56^
Tea (*Camellia sinensis* L.)	−5°C, 3 h	100 µM	MET treatment mitigated the CS-induced reductions in photosynthetic capacity by reducing oxidative stress through enhanced antioxidant potential and redox homeostasis	^57^
Tea (*Camellia sinensis* L.)	4°C	100 µM	MET pretreatment alleviated the ROS burst, decreased MDA levels, maintained high photosynthetic efficiency, and increased the activities of SOD, POD, APX, and CAT	^58^
Rice (*Oryza sativa* L.)	15/15°C	150 μM	MET alleviated low-temperature stress through AB15-mediated signals during seed germination.	^59^
Alfalfa (*Medicago truncatula* L.)	4°C	75 μM	MET pretreatment enhanced the antioxidative ability by improving the activities of POD, SOD, CAT and APX, helping the plants counteract CS-mediated damage by strengthening the non-enzymatic antioxidant system	^48^
Barley (*Hordeum vulgare* L.)	5°C	1 μM	MET pretreatment improved the activities of SOD and CAT and also helped plants sustain stable redox homeostasis	^60^
Pepper (*Capsicum annuum* L.)	5/10 ± 0.5°C, 72 h	5 µM	MET treatment improved water relations, photosynthetic parameters, and antioxidant enzymes’ activities while lowered MDA and H_2_O_2_ contents and membrane permeability	^61^
Strawberry (*Fragaria × ananassa* L.)	0/−4 ◦C (16/8 h), 2 d	100 μM	MET pretreatment protected plants from the cold damages induced through enhanced antioxidant defense potential and modulated the DREB/CBF – COR pathway.	^62^
Pepper (*Capsicum annuum* L.)	15°C/5°C	200 µmol L^−1^	MET alleviated low temperature-induced stress by GA3, IAA, and ZT accumulation while decreased ABA level	^63^
Eggplant (*Solanum melongena* L.)	5°C/10°C (night/day), 3 d	1, 5 or 25 μM	MET alleviated adverse effects of chilling stress, and increased the APOX, POX, CAT, and photosynthetic activities	^64^
Banana (*Musa* spp.)	4°C	50, 100, 150 and 200 μM	MET improved the electron transfer rate, total antioxidant capacity, CAT and SOD activities and proline and soluble sugar contents and significantly reduced the accumulations of MDA, superoxide anion and H_2_O_2_	^65^
**Heat stress**				
Perennial ryegrass (*Lolium perenne* L.)	38/33°C	20 μM	MET treatment alleviated growth inhibition and leaf senescence, increasing the melatonin and CK content’s endogenous content and decreasing ABA content. It also up-regulated the CK biosynthesis genes expression, while the biosynthesis and signaling genes involved in ABA were down-regulated	^66^
Kiwifruit (*Actinidia deliciosa*)	45 ◦C	200 µM	MET alleviated heat-induced oxidative harm through reducing H_2_O_2_ content and increasing proline content, raised ascorbic acid levels, and the activity of antioxidant enzymes, including SOD, CAT, and POD	^67^
Tall fescue (*Festuca arundinacea*)	42°C	1 mM and 50 mM	MET treatment decreased ROS, electrolyte leakage, and MDA but increased Chl, total protein, and antioxidant enzyme activities	^68^
Tomato (*Solanum lycopersicum* L.)	28 ± 1°C, 36 h	100 μM	MET pretreatment reduced the oxidative stress by controlling the over-accumulation of O_2_^•−^ and H_2_O_2_, lowering the lipid peroxidation content and less membrane injury index	^69^
Tomato (*Solanum lycopersicum* L.)	40°C, 9 h	10 μM	Endogenous MET alleviated the heat-induced oxidative stress by maintaining an efficient enzymatic antioxidant system and redox homeostasis.	^70^
Radish (*Raphanus sativus* L.)	35°C/30°C day/night	0, 11.6, 17.4, 29.0, 34.8 and 67.0 mg L^−1^	MET treatment increased the antioxidant enzyme activity, APX, Chl a, and carotenoid contents compared with the control. The auxin and ABA contents were also increased significantly	^71^
Wheat (*Triticum aestivum* L.)	42 ◦C	100 µM	MET treatment reduced oxidative stress by preventing the over-accumulation of H_2_O_2_, lowering lipid peroxidation, MDA, and increasing proline biosynthesis. It also increased the activities of antioxidant enzymes, such as SOD, CAT, and POD	^72^
*Pinellia ternata*	35°C day/30°C	100 μM	MET treatment increased Chl content and relative water content and decreased MDA and electrolyte leakage. Also, activated *HSPs*, ribosomal proteins, and ROS-scavenging enzymes	^73^
Tall fescue (*Festuca arundinacea*)	42°C	20 µM	MET reduced the heat-caused damaging effects on Chl a, Chl b, carotenoid, and protein synthesis machinery. It also enhanced the activities of antioxidant enzymes, protein, and lipid molecules and favored the lower production of H_2_O_2_ and MDA	^74^
Strawberry (*Fragaria × ananassa* Duch.)	35°C and 40°C	0, 50, and 100 μM	MET treatment decreased heat injury symptoms and induced antioxidant mechanisms, also up-regulated the expression of defense HSF (*FaTHsfA2a, FaTHSFB1a*) and HSP (*HSP90*) genes	^75^
Rice (*Oryza sativa* L.)	38 ◦C	0, 20, 100, 500 µM	MET treatment improved the heat tolerance of rice seeds by enhancing the activity of the antioxidant enzymes and significantly reducing the MDA content	^76^
Tomato (*Solanum lycopersicum* L.)	40°C, 7 d	50 mM	MET alleviated the oxidative damage of PSII by balancing the electron transfer of the donor side, reaction center, and receptor side	^77^
Rice (*Oryza sativa* L.)	38°C/28°C	250 mL of 200 μmol L^−1^	MET improved the stress resistance by enhancing the scavenging efficiency of ROS and improved the leaf photosynthetic and heat-resistance properties.	^78^

Abbreviations: Abscisic acid (ABA); ascorbate peroxidase (APX); cold stress (CS); cytokinin (CK); chlorophyll (Chl); catalase (CAT); glutathione reductase (GSSG-R); gibberellic acid (GA3); heat stress (HS); hydrogen peroxide (H_2_O_2_); heat shock proteins (HSPs); indole-3-acetic acid (IAA); malondialdehyde (MDA); melatonin (MET); peroxidase (POD); reactive oxygen species (ROS); superoxide dismutase (SOD); zeatin (ZT).

Under CS, treatment of *D. odorifera* seedlings with exogenous MET and Ca^2+^ (0.6 mM + 5 mM CaCl_2_) improved the plant growth and relieved injuries.^79^ Exogenous MET and Ca^2+^ enhanced photosynthetic and antioxidant activities and decreased lipid peroxidation, electrolyte leakage, and ROS generation under CS (3°C). In addition, exogenous MET and Ca^2+^ intensely raised the phytohormones levels like gibberellin (GA3) and auxin (IAA); however, abscisic acid (ABA) was decreased. The GA3 and IAA contents were improved respectively by 22% and 17% in seedlings when treated with combined MET-Ca^2+^ than MET alone.^79^ In a study, hulless barley seeds were grown under different temperatures (25°C, 15°C, and 5°C) and treated with MET (1 µM) solution for 12 hours before the seeds germinated.^80^ Exogenous MET (1 µM) treatment alleviated the growth inhibition caused by CS and also restored the circadian rhythmic oscillation of circadian clock genes *HvCCA1* and *HvTOC1*, whose circadian rhythmic phenotypes were lost by CS. The findings confirmed that MET treatment also lessened the soluble sugars and malondialdehyde (MDA) contents.^80^ Pepper seedlings and flowering were pre-treated with 5 µM MET and then were exposed to CS at 5°C/10°C (night/day) for 3 days.^61^ The results revealed that MET treatment improved CS tolerance by improving photosynthetic parameters, water relations, and antioxidant enzyme activities while lowering H_2_O_2_ and MDA contents and membrane permeability.^61^ In another study, the exogenous role of MET was investigated on soybean seedlings when exposed to CS.^81^ The results revealed that CS enhanced oxidative damage by ROS accumulation, affecting the growth and development of soybean. However, 5 µmol L^−1^ MET treatment alleviated the oxidative damage by increasing the transcript abundance of antioxidant-related genes.^81^

A recent study investigated the effect of 100 μM MET on bell pepper while storing at 4°C for 20 days and afterward shelf at 20°C for 3 days.^82^ The findings revealed that MET treatment lessened the cell structure damage and lightened the increase in chilling injury incidence, MDA content, and membrane permeability under CS conditions. Moreover, MET activated the antioxidant defense system to fight oxidative damage by up-regulating the expression levels of *CaSOD, CaPOD, CaCAT*, and *CaAPX* genes.^82^ During cold storage of pomegranate fruit at 4°C for 120 days, different doses of MET (0, 1, 10, 100, and 1000 μM) confer CS tolerance.^83^ The results confirmed that treatment with MET attenuates the H_2_O_2_ accumulation by improving GR, APX, CAT, and SOD activity, maintaining membrane integrity by hindering LOX and PLD activity. Therefore, exogenous MET is a safe strategy for providing CS tolerance in pomegranate fruit.^83^

Cut anthurium flowers could develop chilling injury during low-temperature storage, manifested as spathe browning.^84^ MET treatment (1, 10, 100, and 1000 μM) ameliorated chilling injury in cut anthurium flowers by 11, 29, 51, and 31%, respectively, than untreated flowers. Furthermore, MET triggers the proline synthesis and enhances ROS avoiding and scavenging activity, increasing GR, CAT, APX, and SOD activities.^84^ In another study, the authors studied the impact of exogenous MET treatment on chilling injury in tomatoes during cold storage.^85^ They found that 100 μM of MET application alleviated chilling injury and provided enough intracellular ATP by higher H^+^-ATPase, Ca-ATPase, cytochrome C oxidase (CCO), and succinate dehydrogenase (SDH) enzyme activity. It also protected membrane integrity due to a higher unsaturated/saturated fatty acids ratio as a consequence of higher *FAD3* and *FAD7* genes expression, which coincided with lower *PLD* and *LOX* genes expression and enzyme activity.^85^ Cold stress decreased the biomass, photosynthetic pigments, and mineral nutrients of *Medicago truncatula* plants. The authors studied that exogenous MET and *Rhizobium* inoculation (RI) attenuated CS-induced injuries and reduced oxidative damage.^48^ MET pretreatment also enhanced the antioxidative ability by improving the activities of APX (42%), CAT (140%), SOD (50%), and POD (8%), also increased osmolyte accumulation, nutrient uptake, and nitrate reductase activity.^48^

Recent studies also showed that MET plays a vital role in maintaining the fruit’s health during cold storage. For instance, during cold storage at 0°C for 45 days, sweet cherry fruits were treated with different MET levels (1, 10, 100, and 1000 µM). MET treatment retarded the fruit senescence and improved the antioxidant potential.^86^ The results showed that 1000 µM of MET treatment exhibited the lowest flesh browning and decayed incidence after 45 days of storage at 0°C. Moreover, exogenous MET enhanced the activities of APX, SOD, GR, and CAT while lowered the activities of lipoxygenase and phospholipase D.^86^ Another study demonstrated that the plum fruit treatment with 1.0 mmol L^−1^ MET mitigated the fruit chilling injury by reducing flesh-reddening and ‘ethylene burst.’ The MET application also repressed fruit softening and maintained energy status. In contrast, MET reduced the accumulation of cold-induced secondary metabolites.^87^ Likewise, MET application in kiwifruit alleviated chilling injury during cold storage.^88^ MET treatment strongly repressed the activity of lignin metabolism enzymes (PAL, 4CL, and C4H) and the expression of structural genes, whereas improved the activity of antioxidant enzymes (SOD, CAT, APX, and GR) and the accumulation of antioxidant substances (AsA and GSH). The outcomes propose that MET actively participates in cold tolerance and lignin accumulation through enzyme activity regulation in postharvest kiwifruit.^88^

Another study evaluated the influence of MET application on the chilling injury and quality of guavas during storage at 4 ± 1°C.^89^ MET treatment showed lower weight loss, cell membrane permeability, and chilling injury index also delayed the decreases of fruit firmness, sucrose, total soluble sugar, vitamin C, titratable acidity, and total soluble solids. These findings suggest that MET treatment could improve chilling tolerance and retain the quality of cold-stored guavas.^89^ Likewise, MET treatment confers chilling tolerance in mango fruit.^90^ The MET application increased polyamine accumulation and GABA shunt pathway activity.^90^ MET application also increased the accumulation of endogenous polyamines such as ADC and ODC and lowered DAO and PAO activities in the peel and pulp.^90^ In a recent study, the authors investigated the effects of exogenous MET on the ripening and decay incidence of plum fruit.^91^ The results presented that MET could slow the ripening process as directed by the firmness, respiration rate, and ethylene production and reduced the weight loss and decay incidence of plum fruit in room storage.^91^ In addition, MET application triggered the phenylpropanoid pathway by enhancing the activities of phenylalanine ammonia-lyase (PAL), 4-coumarate-coenzyme A ligase (4CL), cinnamate-4-hydroxylase (4CH) and peroxidase (POD), and accompanied by higher contents of total phenols and lignin, which might be contributed to improving the temperature tolerance in plum fruit during storage.^91^ In a nutshell, all these examples suggest that exogenous MET significantly improves CS tolerance by regulating several physiological, biochemical, and molecular mechanisms in different fruits and crop plant species.

### 3.2. Heat Stress

Heat stress (HS) is one of the major limiting factors for plant growth and causes a severe decline in global crop production. Heat stress accelerates crop senescence in extreme environments, decreasing crop productivity and even may cause death.^4–6,92,93^ HS also disturbs the respiration and photosynthesis-related metabolic processes leading to disruption of the carbon economy of the plants under HS condition (Fig. 3).^93–95^ MET acts as a plant growth regulator and plays an essential role in maintaining many physio-biochemical and molecular processes to cope with HS conditions (Table 1).

Pretreatment of tomato seedlings with 100 μM MET and then were exposed to HS (42°C) for 24 h.^69^ After heat shock, the molecular analysis showed that MET treatment effectively lessened the oxidative stress by regulating the over-accumulation of superoxide (O_2_^•−^) and hydrogen peroxide (H_2_O_2_), reducing the lipid peroxidation content and less membrane injury index. So, MET alleviated HS injuries by improving antioxidant defense mechanisms, such as the ascorbate–glutathione cycle, and reprogramming the NO biosynthesis and PAs metabolic pathways.^69^ Cherry radish was cultured at 35°C/30°C day/night high temperature and applied different MET concentrations, i.e., 0, 11.6, 17.4, 29.0, 34.8, and 67.0 mg L^−1^.^71^ The results showed that MET treatment significantly increased cherry radish biomass by 13%, and the soluble solid and soluble protein were improved by 9% and 18%, respectively. MET treatment also enhanced the activities of antioxidant enzymes, ascorbate, Chl a, carotenoid contents, ABA, and auxin contents. Thus, MET application positively affected cherry radish growth under HS.^71^

In a recent study, it was found that MET promotes photosynthesis and biomass accumulation in tea plants and improves tea quality under HS.^96^ MET treatment reduced the polyphenol to free amino acid ratio by finely refining the concentrations of polyphenols and amino acids. The qRT-PCR analysis discovered that MET amplified the transcript levels of catechins biosynthesis genes, including *CsCHS, CsCH1, CsF3H, CsDFR, CsANS, CsLAR*, and *CsANR*, under HS.^96^ Pretreatment of wheat seedlings with 100 µM MET followed by exposure to HS resulted in efficiently reduced oxidative stress by avoiding the over-accumulation of H_2_O_2_, dropping the MDA content, and growing proline biosynthesis.^72^ Moreover, the activities of antioxidant enzymes, like SOD, CAT, and POD, were increased substantially. Furthermore, the expression of ROS-related genes *TaSOD, TaPOD*, and *TaCAT*, and anti-stress responsive genes, such as *TaMYB80, TaWRKY26*, and *TaWRKY39*, were also induced in MET-treated seedlings.^72^

In *Mentha × piperita* L. and *Mentha arvensis* L. plants, a group of researchers assessed the chemical profile changes of essential oil and antioxidant enzymes activity in response to HS.^97^ The findings revealed that MET treatment plays an essential role in regulating physiological processes and alleviating HS. External MET applications also triggered a significant rise in enzymatic antioxidants, including SOD, POX, CAT, and APX peroxidase, and non-enzymatic antioxidants like ascorbic acid and vitamin E, causing reduced ROS levels and lipid peroxidation under HS.^97^ In another study, it was reported that irrigation treatment with 20 µM of MET significantly alleviated HS-induced pollen inactivation in tomatoes.^98^ MET treatment alleviated the ROS synthesis by up-regulating the transcription and activities of antioxidant enzymes under HS conditions. The results suggest that MET protects pollen activity and reduces ROS accumulation by inducing antioxidant enzyme activities under HS.^98^ In a recent study, regulatory networks of MET in chrysanthemum seedlings were explored in response to HS.^99^ The findings revealed that MET treatment decreased the H_2_O_2_ content and MDA accumulation, promoting the proline and soluble protein, GSH, and AsA accumulation, and antioxidant enzymes activities, including SOD and POD CAT, and APX under HS. Moreover, MET improved the dry weight, fresh weight, photosynthesis rate, Chl content, and gas exchange parameters.^99^

In a recent study, the authors found that 100 μM of MET application enhanced tomatoes’ CO_2_ assimilation and photosynthetic pigment content under HS. MET protects the PSI and PSII reaction center and lessens photo-inhibition.^100^ Likewise, HS (42°C) enhanced the ROS and lowered the photosynthesis efficiency in soybean.^101^ While pretreatment with 100 µmol of MET improved the photosynthetic pigment (Chl a and Chl b), plant growth and reduced the oxidative stress via scavenging superoxide and H_2_O_2_ and reducing the electrolyte leakage and MDA contents.^101^ A recent study with meta-analysis revealed that MET treatment improves the maize root length, plant height, leaf area, shoot weight, fresh and dry root weight, CAT, POD, SOD and APX activities, protein, and soluble sugar under stress conditions.^102^ In contrast, MET treatment lowered the levels of H_2_O_2_, O_2_^−^, MDA, and electrolyte leakage. In conclusion, MET relieves oxidative damage by refining stress tolerance, regulating the antioxidant defense system, and improving leaf Chl content compared to control.^102^ The effect of MET priming on the photosynthetic electron transport of PSII against heat stress was evaluated in tall fescue. The results revealed that MET weakened the electron transfer efficiency of PSII per light reaction center at the receptor and donor sides. In contrast, it improved the number of reaction centers per unit cross-sectional area. In brief, MET regulates the photoelectric conversion of PSII of tall fescue under heat stress and improves its survival rate after heat shock.^103^ In conclusion, exogenous MET treatment improves plant growth and development by inducing the antioxidant defense mechanisms, interacting with other chemicals, and reprogramming the biochemical metabolism.

## 4. Melatonin-mediated Genetic Engineering in Improving Temperature Stress Tolerance in Plants

### 4.1. A Brief Overview of Genetic Engineering in Plants

Biotechnology has given marvelous and fantastic scope for crop development, crop protection, crop quality management, and enhancing other agricultural attributes.^104^ In certain plants, genetic engineering has allowed for the integration of specifically desired features. Isolating a gene of interest, ligating that gene with a suitable vector to generate a recombinant-DNA molecule, and then transferring that gene into the plant genome to develop a novel function are all examples of genetic engineering.^104,105^ Transgenic technology has been dubbed “agriculture’s fastest expanding technology.” It refers to a collection of approaches for transferring desirable gene(s) across taxonomic boundaries into a specific plant via non-conventional means from any source (plants, animals, microbes, or even artificially generated genes).^106,107^ The cornerstone and essence of sustainable agriculture are combating numerous sorts of abiotic stressors like temperature. The primary benefits of transgenic technology are that the genes regulating many agronomically significant features may be derived from any organism – plants, bacteria, etc., and used to alter plants. Novel features from any background can thus be easily integrated into the target plant.^106,107^

The clustered regularly interspaced short palindromic repeats (CRISPR)/associated protein 9 (Cas9) system is a valuable genome editing tool having specificity, high efficiency, and processing a wide range of applications.^108^ CRISPR/Cas9 is more rapid, less expensive, precise, and highly effective in editing genomes even at the multiplex level than other genome editing technologies like transcriptional activator-like effector nucleases (TALENs) and zinc finger nucleases (ZFNs). CRISPR/Cas9 has shown the most significant promise for addressing emerging challenges in agriculture.^109^ Targeted knock-out/in, substitution, insertion, and deletion mutations created by CRISPR/Cas9 system have explored the regulatory functions of genes and their impact on other biochemical processes and helped to improve crops under abiotic stresses by their scavenging capacity.^108^ CRISPR/Cas9 can be easily programmed to include double-strand breaks at any desired target site. CRISPR/Cas9-based genome editing helps to create novel cultivars with desirable traits such as yield improvement, enhancement in yield quality and higher resistance to biotic and abiotic stresses.^110^ Moreover, through this genetic engineering methodology, genetic breeders can eliminate unfavorable traits or add favorable traits in a straightforward process needing only one single generation.^108^ Recently, CRISPR/Cas9-based genome editing have been widely utilized to improve temperature tolerance in different plant species.^111–115^

### 4.2. Melatonin Enhances Temperature Tolerance in Transgenic Plants

Genetic engineering of MET-encoding genes is a fascinating area that can also help improve the temperature stress tolerance in plants. However, only a few studies have looked at the effect of MET in heat-stressed crops at the genetic/molecular level. MET increased the production of heat shock proteins in tomato cells in response to HS, which controlled cell structure and protects cellular protein structure and stability under HS.^116^ Tryptophan decarboxylase (*TDC*), the first gene involved in MET production, has been overexpressed in rice. MET buildup in *TDC3* transgenic lines is seed-specific; transgenic seeds had 31-fold greater MET concentrations than wild-type seeds.^117^

High temperatures and dark conditions increased MET levels of the final two enzymes in the MET production process.^118^ This suggests that MET has a function in temperature regulation. Microarray examination of global gene expression patterns in transgenic rice seedlings overexpressing the sheep *SNAT* gene resulted in an endogenous rise in MET levels relative to the wild type.^119^ Because of the increased levels of endogenous MET, the MET-rich transgenic rice plants showed higher oxidative stress resistance against cold. According to the microarray analysis, the endogenous MET increase in rice resulted in a moderate level of gene expression changes, with 464 genes differentially expressed.^120^ According to a previous report, genome-wide transcriptome analysis showed that nearly 4000 transcripts were differentially expressed as a result of MET exposure in transgenic bermudagrass (*Cynodon dactylon*). MET had many favorable impacts on transgenic bermudagrass under CS (4°C).^121^ Moreover, it was reported sheep (*Ovis aries*) SNAT (*OaSNAT*) gene is expressed in tobacco leaves. *OaSNAT* was found to be transiently expressed and exhibited a bright red fluorescence, indicating that the animal’s OaSNAT protein was expressed in the plant under HS (30°C).^119^

A previous study discovered that the development of ubiquitinated protein in the insoluble protein aggregates of MET treated and *ASMT* overexpressed tomato plants was greatly reduced compared to WT plants after 9 hours of HS (40°C) treatment.^116^ These results imply that either exogenous MET or endogenous MET manipulation via *ASMT* overexpression decreases HS-induced protein aggregation and ubiquitination. Heat stress significantly increased transcript levels of *HSP17.4, HSP20, HSP20-1, HSP21, HSP70*, and *HSP90* in WT plants within 3 hours. More importantly, the expression of these HSPs was increased in MET-treated and *ASMT* plants following HS, implying that MET activated HSPs to aid in the refolding of denatured proteins.^116^ Under HS (28°C), MET concentrations in transgenic rice seeds were 31 times greater than those in WT seeds. Both in homologous and ectopic *TDC* overexpression lines, the level of MET intermediates was likewise elevated and improved the HS tolerance in transgenic rice lines.^122^ In plants treated with MET, the relative normalized expression of *COR15a* was seven times greater, and a four-fold induction was seen in MET-treated plants after 0.5 hours of CS treatment.^123^ These results suggest that the up-regulation of CS-related genes in transgenic plants by MET may stimulate the biosynthesis of cold-shielding compounds and improve plants growth treated with exogenous MET under CS.

Interestingly, MET treatment also plays key role in gene expression and cell suspension under temperature stress. For instance, it was reported that pIPKb002-TDC3 binary vector was used to create the *TDC3* transgenic rice line, which was able to synthesize MET when compared to three separate transgenic rice plants that expressed each *TDC* isogene separately for MET production.^117^ While the WT and other *TDC* genotypes showed considerable expression of *TDC* under HS (28°C), there was specific *TDC* overexpression for each TDC isogene.^117^ In another study, preincubation with MET prior to CS treatment (2–3°C) protected carrot suspension cells from dying, as detected by trypan blue staining.^124^ When cells were preincubated with 86 nm MET before being exposed to cold, there was only a small drop in cell viability, after a 7-day CS, the proportion of TUNEL-positive cells reduced from 83.6 to 12.8% as MET concentrations increased.^124^

Furthermore, a previous study found that when tomatoes were subjected to a drought treatment at 20°C, WT plants lost water faster than transgenic ‘Micro‐Tom’ tomato overexpressing ovine *AANAT* and ovine *HIOMT* genes.^125^ The average mass ratio of transgenic lines declined from 100% to around 65% after 4 hours of HS treatment. This is substantially higher than the WT, which experienced a reduction from 100% to 50%. In fact, the three transgenic lines had a higher mass ratio than the WT at all times during HS (20°C) treatment, and transgenic plants overexpressing ovine *HIOMT* were more drought tolerant.^125^ According to scientists, at – 20°C, MET triggered the synthesis and breakdown of cellulose, pectin, and xylan, suggesting that MET may alleviate the effects of temperature stress based on the trials above.^30^ Previously, it was opined that the leaves of WT and *snat* mutant lines were inoculated with Pst-avrRpt2 to see how decreased MET production caused by the knockout of *SNAT* gene function affected pathogen resistance in *Arabidopsis thaliana* under HS (28°C).^126^ The knockout mutant lines were more susceptible to pathogen assault than WT plants, suggesting the direct involvement of MET as a signal molecule in pathogen resistance, mainly under HS conditions.^126^

Through the dual regulation of leaf senescence and vascular development, a recent study discovered the involvement of a caffeic acid O-methyltransferase (*OsCOMT*) gene in modulating rice grain yield.^127^ The results revealed that *OsCOMT* is involved in the production of MET.^127^ According to transgenic assays, *OsCOMT* dramatically slowed down the senescence of leaves at the grain-filling stage by preventing the breakdown of Chl and chloroplast, which boosted the effectiveness of photosynthesis. Briefly, MET-mediated leaf senescence and vascular development offer a potential method for genetically enhancing rice grain output.^127^ In watermelon, 16 putative O-methyltransferase (*ClOMT*) genes were identified. Among them, *ClOMT03* (*Cla97C07G144540*) was considered a potential *COMT* gene (renamed *ClCOMT1*) based on its high identity (60.00–74.93%) to known *COMT* genes involved in MET production, expressed in nearly all tissues, and upregulation during abiotic stressors.^128^ Watermelon calli with *ClCOMT1* overexpression had considerably higher MET concentrations than those with *ClCOMT1* deletion using the CRISPR/Cas-9 technology. These results suggest that *ClCOMT1* plays an essential role in MET biosynthesis in watermelon. Cold, drought and salt stress also increased MET concentrations while up-regulated *ClCOMT1* expression in watermelon. *ClCOMT1* is a positive regulator of plant tolerance to abiotic stimuli, as shown by the improved tolerance of transgenic *Arabidopsis* to such challenges when *ClCOMT1* was overexpressed.^128^ So far, MET-encoding genes have not been widely edited using CRISPR/Cas system; therefore, more efforts are required to apply the genome editing application to fully unravel the MET role against temperature stress. The use of CRISPR/Cas9 for modifying plant genomes is fast growing. To offset the harmful effects of climate change and secure the future food security of growing populations in tropical nations, the CRISPR/Cas9 system is evolving into a user-friendly tool for the generation of non-transgenic genome-edited crop plants.

## 5. Melatonin Interaction and Crosstalk with Other Molecules under Temperature Stress

Melatonin (MET) acts as a multifunctional molecule that is widely involved in plant growth, development and stress responses. Numerous studies reveal that MET interacts with other signaling molecules, including ROS, nitric oxide (NO) and hydrogen sulfide (H_2_S).^22,129–131^ The interactions among MET, ROS, NO and H_2_S demonstrate the responses to abiotic stress through a series of signaling networks, which confer the plant adaption and tolerance. Moreover, MET is able to interact jointly with H_2_O_2_ and NO, resulting in post-translational changes and regulation of hormonal activities.^129–132^ There is also established crosstalk between MET and phytohormones such as auxin, cytokinin, gibberellins, ethylene and ABA.^133^ In this section, we demonstrated the significance of MET interaction, regulation of redox homeostasis via antioxidant system, and protection of plants from oxidative stress-induced cellular injuries in response to temperature stress.

**Figure 5.**
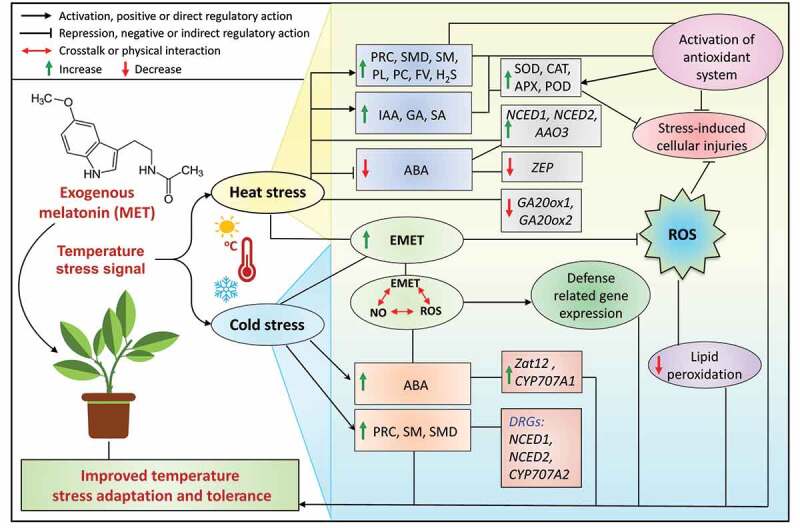
Interaction between melatonin and other biomolecules in plants under temperature stress conditions. Exogenous MET-mediated induction of exogenous MET and other biomolecules occurs in response to heat stress, which inhibits abscisic acid induction. The cold stress shows the opposite effect on abscisic acid regulation compared to heat stress in plants. Several candidate genes respond differently to cold and heat stresses, particularly defense-related genes, which help to plant stress tolerance. Endogenous MET inhibits superoxide molecules, and there is some interaction between endogenous MET, nitric oxide, and reactive oxygen species. Melatonin activates the antioxidant system during stress by increasing antioxidants; the combined mechanisms help to reduce lipid peroxidation, inhibit oxidative stress-induced cellular injury, and improve plant temperature stress tolerance. On the left side, the small chemical structure represents MET. Abbreviations: MET, melatonin; EMET; endogenous melatonin; ABA, abscisic acid; GA, gibberellic acid; IAA, indole-3-acetic acid; SA, salicylic acid; SOD, superoxide dismutase, CAT, catalase; APX, Ascorbate peroxidase; POD, peroxidase; NO, nitric oxide; ROS, reactive oxygen species, PRC, putrescine; SMD, spermidine; SM, spermine; PL, proline, PC, phenolic compound; FV, flavonoid; H_2_S, hydrogen sulfide; DRGs, differentially regulated genes. *LpZEP, LpNCED1, CsZat12, CsCYP707A1, CsNCED1, CsNCED2*, and *CsCYP707A2* genes regulated in response to MET under temperature stress.

Under stressed conditions like HS and CS, the interplay between hormones and MET has been widely reported, which is interrelated and mitigates the damages caused by these adverse conditions.^26,31,36,45^ Plants respond to the temperature stress by using multiple signal pathways, in which MET plays a key role and works synergistically and/or antagonistically with phytohormones as well as other molecules for regulating plant growth, development and defense response (Fig. 5). For instance, an experiment was carried out in which rice plants were subjected to different temperature regimes.^59^ To ameliorate the damages caused by HS, the exogenous supply of MET played a significant impact on plant adaption and stress tolerance.^59^ Exogenous MET treatment altered the NO level, while NO influenced the endogenous MET content.^59^ The interplay of MET, NO and ROS induces diverse physiological behaviors in plants through interaction mechanisms.^129,130^ MET can interact synergistically or antagonistically with phytohormones and regulates their endogenous levels, and these interactions lead to enhancing plant defense under stressful condition. Induction of endogenous phytohormones or MET level due to application or presence of exogenous MET may regulate differentially in different plant species. In the following sections, we updated the studies on how the exogenous supply of MET regulates the level of phytohormones and plant adaption.

Tomato is one of the most important horticultural crops worldwide, and both HS and CS reduce the optimal physiological performance of tomatoes.^134^ In this sense, it was noted that the foliar spraying of tomato plants with 80 mL of 100 μM MET each day for one week before exposure to HS (42°C) for 24 h.^69^ After HS, MET-treated plants showed an increase in the endogenous levels of several polyamines such as putrescine, spermidine, and spermine. Moreover, the findings revealed an increase in NO content.^69^ Working on the same crop, the foliar sprays with 100 μM MET in tomato plants subjected to HS (38/28°C and photoperiod of 16/8 h) for 5 days resulted in a decline in ABA concentration in tomato leaves.^135^ The mRNA levels of several genes such as *NCED1, NCED2*, and *AAO3* involved in ABA biosynthesis were assessed, showing a significant increase under HS and MET application.^135^ On the contrary, GA content in leaves was elevated under HS with MET application. The determination of relative expression of GA biosynthesis genes (*GA20ox1* and *GA20ox2*) were downregulated under HS, while the responses were reversed after the addition of MET supplementation with HS conditions in tomato plants.^135^

According to recent reports, there are other studies focused on the amelioration of exogenous feeding with MET in temperature-stressed plants.^101^ The irrigation with a volume of 30 mL of 100 µM MET twice daily for 6 days in soybean plants subjected to HS (42°C) for 3 and 7 days resulted in an increase in proline, phenolics compounds, and flavonoids concentration as well as in polyamines such as spermine, spermidine, and putrescine.^101^ Moreover, the application of MET reduced ABA content whereas increased the level of salicylic acid.^101^ On the other hand, a study reported that the supply of 100 µM MET under HS in wheat seedlings resulted in an overaccumulation of proline concentration.^72^

The ameliorative effect of exogenous application of MET under HS has also been reported in perennial ryegrass (*Lolium perenne* L.).^66^ This positive effect can be related to the decrease in ABA synthesis, mainly ascribed to the down-regulation of two ABA biosynthesis genes (*LpZEP* and *LpNCED1*). Though, a study found an increased level of auxin and ABA in cherry radish subjected to HS and an exogenous supply of MET.^71^ This study is a bit exceptional in terms of ABA induction in response to MET-mediated HS. Moreover, they reported a positive effect of MET in temperature-stressed plants.^71^ Regarding other biomolecules, the exposure of wheat plants to HS (40°C) for six hours for 15 days was ameliorated by the exogenous application of MET, which led to an increase in hydrogen sulfide contents.^136^ Regarding the interaction with ABA, it was also reported that the exogenous application of MET under CS in *Elymus nutans* resulted in an increase in ABA synthesis.^53^

Under CS, the exogenous MET (200 µM) in cucumber seedlings led to an increase in the polyamines contents (such as putrescine, spermine, and spermidine) and also modulated the expression of the key ABA biosynthesis genes (*CsNCED1* and *CsNCED2*) as well as genes involved in ABA catabolism (*CsCYP707A1* and *CsCYP707A2*).^56^ A recent study reported that CS significantly reduced root length, plant height, leaf surface area, and Chl contents, and increased the ROS levels that led to induced oxidative damage, lipid peroxidation and electrolyte leakage. However, these parameters were significantly reverted after supplementation of MET enhanced stress tolerance.^137^ Recently, it has been reported that there is a correlation between MET with MeJA, which modulate H_2_O_2_ levels and increase CS tolerance in watermelon.^138^ In short, all these examples suggest that MET interacts and crosstalk with hormones, polyamines, and other gaseous molecules to improve temperature stress tolerance by regulating several physio-biochemical and molecular mechanisms in plants. In the near future, more in-depth studies should be carried out to fully reveal the mode of action that can help plants to cope with temperature stress conditions.

## 6. Conclusion and Outlooks

Temperature stress (both heat and cold stress) causes multiple problems at the molecular and physiological levels in plants, resulting in massive productivity losses worldwide. MET is a nontoxic indolic molecule that plays important roles in animals (circadian rhythm, antioxidant activity, and immunity) and plants (photosynthesis enhancement, biomass generation, and plant osmoregulation under abiotic and biotic stressed conditions). The current review has focused on the metabolism and biosynthesis of MET in plants, the beneficial effects of the exogenous supply of MET in crops exposed to low and high temperatures, the recent engineering techniques in melatonin biosynthesis in crops as well as the interaction of this molecule with other hormones under changing temperature conditions. Exogenous MET application improves plant growth under temperature stress by maintaining membrane integrity, improving photosynthetic pigment synthesis, and increasing water and nutrient uptake. MET treatment alleviates the cold-induced osmotic imbalance by enhancing the accumulation of hormones, osmolytes, and secondary metabolites. MET mitigates the detrimental effects of heat stress on the photosynthetic machinery of plants. The production of MDA and H_2_O_2_ was lowered in MET-treated plant seedlings under heat stress, signifying the positive modulation of the antioxidant enzyme defense system. MET effectively scavenges a variety of ROS that protects cells and tissues of organisms from the deteriorated effects of oxidative stress.

MET works synergistically and/or antagonistically to phytohormones as well as other molecules for regulating plant growth, development, and defense response. MET crosstalk and interacts with hormones, polyamines, and gaseous molecules to mitigate the damages caused by temperature stress. The current research outcomes specify that MET, H_2_S, Ca^2+^, H_2_O_2_, NO, and other signaling molecules and the MAP kinase cascade pathway are essential in MET-induced plant tolerance to stress. In addition, MET-mediated genetic engineering could provide a promising approach to unraveling the molecular basis of temperature stress tolerance. Genetic modifications can also improve the MET synthesis in transgenic plants and improve tolerance to temperature stress. Future research is required to know the genetic mechanisms and metabolic pathways involved during recovery under stress conditions on exposure to MET. The information about this topic is vast; nevertheless, there is a complexity of the metabolic pathways and their synthesis in two organelles (chloroplasts and mitochondria) that should be considered. A strong effort is required to elucidate these bottlenecks and provide clarification on several fundamental points. Future research should focus on a deeper understanding of the core MET metabolism by generating overexpressing and knockout mutants via CRISPR/Cas system in different crops of the genes involved in the metabolic pathways.
